# Observations of Change of Climate in Consumption

**Published:** 1835

**Authors:** Henry Huntt


### II. ANALYTICAL.

### Art. I.—Change of Climate in Pulmonary Consumption.

*Observations on a change of climate in Pulmonary Consumption.* By Henry Huntt, M. D.

We have lately been favored, by the respectable Author, with a copy of this little pamphlet, the leading object of which is to inquire into the influence of a change of climate on the prevention and cure of Pulmonary Consumption. Although this malady is less frequent in the valley of the Mississippi, than on the sea-board of the Atlantic, it still is sufficiently common and fatal, to constitute a formidable disease; and our physicians are constantly presented with the difficult questions—"*Doctor, shall I go to the south this fall?*"—"*Will it stop my disease?*" "*Must I visit the Havana?*" "*Will New Orleans answer?*" "*Is there any spot in Florida that is preferable?*" These are momentous questions, and cannot always be answered in a manner that is satisfactory to the patient, or gratifying to the physician.

The object of Dr. Huntt's discourse is to show, by a reference to facts, the correctness of the opinion of our distinguished countryman, Dr. Rush, that the sea-coast is unfavorable to phthisical patients. In doing this, he refers to a variety of authors, who concur in having observed, that a mixture of sea and land air is pernicious to such patients, and that consumption is more frequent in maritime situations than any others.

"In Salem, Mass. during the last five years, it is calculated that the mortality from consumption, is about one-fifth of all the inhabitants that have died.—In Boston, about one sixth.—In New York, about one-fifth.—In Philadelphia, about one-seventh.—In Baltimore, about one-seventh.—In Washington, about one-eighth.—In Charleston, S. C. about one-sixth."

The following extract serves to show, that even the warm and regular atmospheric temperature of the south, is not sufficient to counteract the pernicious influence of a land and sea atmosphere combined.

"Sullivan's Island is very unfavorable to consumptive invalids. Dr. Richard Randall, late a surgeon in the United States' army, who was stationed at different military posts throughout the southern country, states, that to those predisposed to phthisis, the air of Sullivan's Island is highly deleterious in comparison with that of Charleston—that acute pneumonic inflammation is also more prevalent in the former than in the latter place: that the air of the city of Charleston, though otherwise more pure than that of the interior of lower Carolina, is not so well adapted to those predisposed to phthisis: that the inhabitants of Augusta, Savannah, and New Orleans, though possessing fewer advantages of climate, are less obnoxious to pulmonary affections, than those residing in the otherwise comparatively healthy situations on the sea-board. 'Soon after my arrival,' says he, 'at Sullivan's Island, in 1821, as surgeon to that military post, I thought I discovered a stronger predisposition among the soldiers to pulmonary affections of all kinds, than I had observed at any former stations at the south, and my experience during the succeeding winters confirmed this belief; for the proportion of cases of pneumonia, and of deaths from phthisis, were three-fold greater at this post, than at my former stations in the interior of Georgia and Alabama.'

"The bay of St. Louis and Passa Christiana, are also very unfavorable to pulmonary complaints. Col. George Gibson, of the United States' army, who was stationed at the latter place during three summers, informed me that Passa Christiana was a great resort for invalids from every description of disease; and it was a remarkable fact, that the consumptive cases survived but a short time after their arrival there: whereas invalids of every other description recovered most rapidly. This fact is totally subversive of the common opinion relative to the cause of pulmonary affections in the neighborhood of the sea. Passa Christiana is liable to no variety of temperature—its atmosphere is warmed by the gulf stream, and is exempt by distance and the intervening forest from the cold air of the mountains.

"It is evident as we recede from the sea-shore, that the cases of pulmonary consumption become diminished. This disease is scarcely known among the Indians of our forest; and in many parts of the interior of the country, consumption is extremely rare; particularly on the borders of Ohio, Mississippi, and Missouri, where bilious intermittent fevers mostly prevail. Col. Benton, of the United States' senate, from Missouri, informed me, that the climate of that state was peculiarly favorable to consumptive invalids; and this fact is so notorious, that many persons afflicted with this complaint, have removed their residence to Missouri, in order to prolong their lives. It is not unusual in that climate for consumptive invalids to linger a long time; sometimes ten or fifteen years, whereas near the sea-shore it generally destroys life in a few months."

Dr. Huntt has collected a variety of facts from the writers of Europe, to show that the climate of the south of Europe is by no means favorable to consumption, and finally comes to this conclusion:

"After taking this review of the effects of different climates in pulmonary diseases, we are irresistibly led to conclude, that the United States afford advantages more favorable in this complaint than any other country yet known. Here the consumptive invalid has the advantage of every variety of soil and climate—every facility in travelling—mineral waters that are valuable in diseases of the lungs, and above all, the heartfelt satisfaction of enjoying the sympathy, kindness and hospitality of his countrymen. Let us therefore abandon the blind and cruel fashion of sending our consumptive patients to the south of France, Italy, and other foreign countries, where it has been incontestibly proven that the climate is a sure passport to the grave."

Without designating localities, by name, Dr. Huntt expresses himself to the following effect, on the subject of a selection in the United States of residences for the consumptive or predisposed:

"The consumptive invalid should always remove as far from the sea as possible, and take as much exercise as is consistent with his strength, particularly on *horseback;* his diet should be such as is most easily digested—mild and nutricious. High hills or mountains should be carefully avoided; the air of such places is too pure; there the oxygen gas is too stimulating, and is apt to increase the inflammatory action, to suppress the usual expectoration and sometimes to produce hœmoptysis. Low grounds, on the contrary, appear always favorable to consumptive patients; there, the air is of a low quality, and therefore not so stimulating to the lungs. From experiments which have been made in breathing, the hydrocarbonate, and from observation, we are induced to believe that marshy countries, distant from the sea, where bilious intermittent fevers prevail, are also favorable to consumptive invalids. Places where animal putrefaction takes place, are generally favorable to weak lungs."

Our author entertains a high opinion of the Red Sulphur Springs, of the valley of the Kenhawa, in Western Virginia. These, and other springs in that region, have lately attracted the attention of the people of the valley of the Mississippi. The Red Sulphur waters abound in sulphuretted hydrogen gas, but are said by Dr. Cullen, of Richmond, to contain no saline matter. The following extract shows their great influence as a sedative:

"The effect of the water on the pulse is one of the numerous, singular, and powerful properties belongiug to it. In about one-third or one-fourth of the cases in which it is employed, the pulse is reduced from fifteen to thirty strokes in a minute. It lessens arterial action to such a degree, that it seldom fails to remove fever, difficulty of breathing, and pain in the chest—unfortunately it does not lessen the appetite, which being commonly indulged, a fulness of habit rapidly follows its use—this gives rise to increased cough, pain, and spitting of blood. From much observation, I would recommend such patients as cannot restrain the indulgence of their appetite, to remain at home; in such cases, the Red Sulphur Water will do no good, and may do much injury—but when the patient can practice self denial, the water may be taken with *greater advantage in all pulmonary cases, than any other remedy* I have ever seen employed for that purpose. It is also an important remedy in cases of enlarged liver or spleen, in dyspepsia, and in diseases of the mucous membrane generally."

We cannot agree with Dr. Cullen, that this water may be taken with advantage, in "all pulmonary cases." An agent of such antiphlogistic and sedative action, is far from being beneficial, or even safe, in the advanced or suppurating stages of tubercular consumption. In subacute bronchitis, such a remedy would be in place.

For winter residence, Dr. Huntt regards the upland parts of South Carolina, Georgia, and Alabama, especially the pine forests, as the best which the United States can afford. He might have added the interior of Florida as milder, perhaps, than either.

We cannot too strongly insist to all who are interested in the matter, on the great necessity of an *early* change of climate, when such a measure is thought of, either to prevent or cure phthisis. And in this malady, we mean its tubercular variety, is in part the effect of a rigorous and variable climate, all who are predisposed, should make a timely and permanent escape to a milder region. Of what advantage can it be to go south with cavernous lungs, or indurated lungs? The time for action is before disorganization of structure has taken place—afterwards, a little arrest of the fatal stroke is all that can come from flight.

